# Long-term geometric quality assurance of radiation focal point and cone-beam computed tomography for Gamma Knife radiosurgery system

**DOI:** 10.1007/s12194-024-00788-9

**Published:** 2024-03-11

**Authors:** Shingo Ohira, Toshikazu Imae, Masanari Minamitani, Atsuto Katano, Atsushi Aoki, Takeshi Ohta, Motoyuki Umekawa, Yuki Shinya, Hirotaka Hasegawa, Teiji Nishio, Masahiko Koizumi, Hideomi Yamashita, Nobuhito Saito, Keiichi Nakagawa

**Affiliations:** 1 Department of Comprehensive Radiation Oncology, The University of Tokyo, 7-3-1 Hongo, Bunkyo-ku, Tokyo, 113-8655 Japan; 2 Department of Medical Physics and Engineering, Osaka University Graduate School of Medicine, Suita, Japan; 3 Department of Radiology, The University of Tokyo Hospital, Tokyo, Japan; 4 Department of Neurosurgery, The University of Tokyo Hospital, Tokyo, Japan; 5 Department of Neurologic Surgery, Mayo Clinic, Rochester Minnesota, USA

**Keywords:** ICON, Gamma Knife, Accuracy, Radiation focal point, CBCT

## Abstract

To investigate the geometric accuracy of the radiation focal point (RFP) and cone-beam computed tomography (CBCT) over long-term periods for the ICON Leksell Gamma Knife radiosurgery system. This phantom study utilized the ICON quality assurance tool plus, and the phantom was manually set on the patient position system before the implementation of treatment for patients. The deviation of the RFP position from the unit center point (UCP) and the positions of the four ball bearings (BBs) in the CBCT from the reference position were automatically analyzed. During 544 days, a total of 269 analyses were performed on different days. The mean ± standard deviation (SD) of the deviation between measured RFP and UCP was 0.01 ± 0.03, 0.01 ± 0.03, and −0.01 ± 0.01 mm in the *X*, *Y*, and *Z* directions, respectively. The deviations with offset values after the cobalt-60 source replacement (0.00 ± 0.03, −0.01 ± 0.01, and −0.01 ± 0.01 mm in the *X*, *Y*, and *Z* directions, respectively) were significantly (*p* = 0.001) smaller than those before the replacement (0.02 ± 0.03, 0.02 ± 0.01, and −0.02 ± 0.01 mm in the *X*, *Y*, and *Z* directions, respectively). The overall mean ± SD of four BBs was −0.03 ± 0.03, −0.01 ± 0.05, and 0.01 ± 0.03 mm in the *X*, *Y*, and *Z* directions, respectively. Geometric positional accuracy was ensured to be within 0.1 mm on most days over a long-term period of more than 500 days.

## Introduction

Leksell Gamma Knife (LGK) radiosurgery system serves as an alternative to neurosurgery for various intracranial diseases such as malignant and benign brain tumors, cerebrovascular malformations, and trigeminal neuralgia [1–5], and patients do not require general anesthesia and usually receive treatment while awake. The LGK equips approximately 200 radioactive cobalt-60 sources emitting gamma rays, and these gamma rays converge at a radiation focal point (RFP), called the “unit center point (UCP)” whose coordinates are (100.0, 100.0, 100.0) in the LGK coordinate system [6], to deliver a high-dose focused radiation to the target while minimizing radiation damage to surrounding healthy tissue.

To achieve precise dose delivery, patients are immobilized using a lightweight frame (Leksell Coordinate Frame (Elekta AB, Stockholm, Sweden) attached to the head with four pins. The treatment plan needs to be generated based on the stereotactic magnetic resonance images (MRI) or computed tomography (CT) that are taken with the frames attached, and the frames must remain in place until treatment planning and treatment are complete. Thus, patients spend a long time with the frames on, and the burden on the patient is high. The frame enables precise fixation of the patient’s head during the treatment procedure [7]; therefore, a margin for gross tumor volume is not required to compensate for the uncertainty in patient head positioning, in principle [8]. Rigorous geometric quality assurance (QA), confirming that the RFP of the gamma rays corresponds to UCP, is essential for treatment accuracy.

**Fig. 1:**
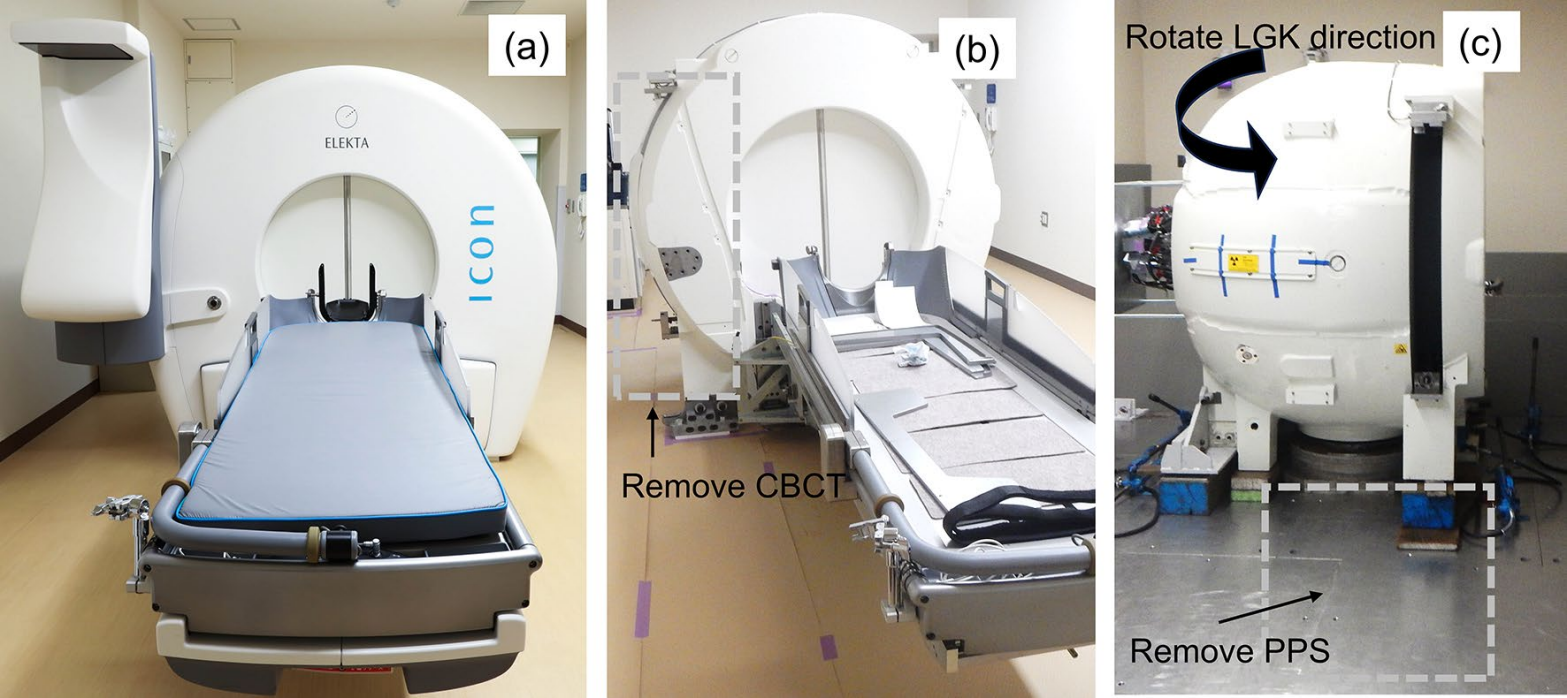
**a** Overview of the LGK system, and **b** the source replacement extensive work to remove the CBCT, and **c** PPS and rotate the LGK unit

The latest clinically available ICON LGK system (Elekta AB) incorporates an on-board cone-beam CT (CBCT), enabling a pre-planning workflow in clinical practice. In the workflow, treatment plans based on the non-stereotactic MRI and CT without frame are generated (pre-plan) before the day of treatment. On the day of treatment, the stereotactic CBCT with frame is acquired and the pre-plan is re-optimized, accounting for the difference in patient position between preoperative non-stereotactic imaging [9]. The time for the patients to attach the frame is shortened markedly, and the patient burden is smaller than in a workflow without CBCT. This workflow also allows frameless treatment using a thermoplastic mask for patient head fixation. On the day of treatment, the masked CBCT is registered to the non-stereotactic imaging (MRI or masked CT), and the pre-plan is re-optimized as in the treatment planning procedure using the frame. These workflows for LGK treatment utilizing CBCT on patients immobilized with a frame or mask can improve patient comfort; however, need to ensure the geometric accuracy of CBCT, and the stereotactic coordinate system established by the CBCT system should be precisely aligned with the Leksell coordinate system. American Association of Physicists in Medicine (AAPM) Task Group (TG) 178 stated that the coincidence of the UCP and the RFP and the alignment of the CBCT should be verified before each treatment using the tools and procedures provided by the manufacturer [6]. The extensive QA work takes a substantial amount of time. If the accuracy of agreement between UCP and RPF is excellent, QA could be simplified, but few papers have investigated the long-term accuracy of UCP and RPF. One concern in simplifying QA is that cobalt-60 sources have a finite half-life time, requiring periodic source replacement operations. Figure 1 shows the overview of the LGK system (a), and the source replacement requires extensive work to remove the CBCT (b) and patient positioning system (PPS) and rotate the LGK unit (c).

This study aims to investigate the geometric accuracy of the RFP and CBCT in relation to the Leksell coordinate system over long periods for the ICON LGK system. Furthermore, the geometric accuracy before and after the cobalt-60 source replacement operation is compared.

## Materials and methods

**Fig. 2:**
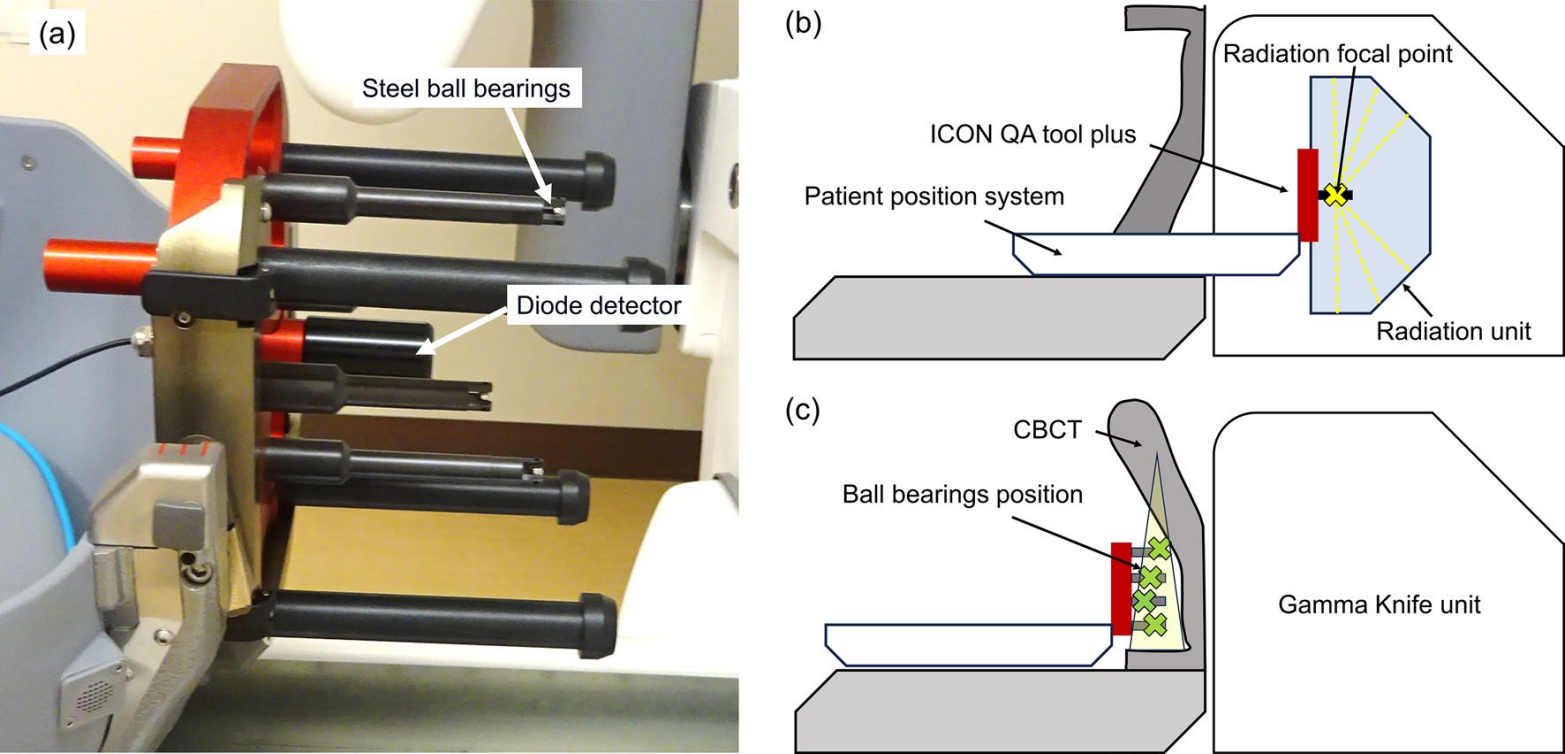
**a** ICON quality assurance (QA) tool plus, and schematic overview of **b** Focus and **c** CBCT precision QA

Ethical approval was not required because our design only involved the use of phantoms. Figure 2 (a) shows an ICON QA tool plus employing a centroid diode detector, and four steel ball bearings (BBs), each with a diameter of 4 mm. These BBs are strategically positioned to ensure that they do not shade each other or the precision diode in the X-ray projection images during CBCT acquisition.

The details of Focus and CBCT precision QA are described in the vendor-provided white paper [10]. Briefly, in the Focus precision QA, the PPS attached to the QA tool plus moves, and the dose profiles through the radiation shot of a 4 mm are scanned using the diode detector in the *X*, *Y*, and *Z* directions (Fig. 2(b)). The respective *X*, *Y*, and *Z* directions indicate the left–right, anterior–posterior, and superior–inferior directions in the LGK coordinates for a supine patient. The positive value in each direction represents the left, posterior, and superior directions. For each profile, the 47%, 50%, and 53% positions relative to the profile’s peak on both rising and falling edges were determined. By repeating this process in reverse, 12 positions in each coordinate direction were obtained, and their average is determined as RFP. The difference in position between measured RFP and UCP is calibrated as the offset values by a service engineer periodically maintenance (once a half year or after replacement of cobalt-60 sources). In the treatment, the treatment plans are adjusted for the known offset to coincide the RFP with the UCP. In the CBCT precision QA (Fig. 2(c)), the positions of BBs in the X-ray projection image during CBCT acquisition are automatically detected, and the BB positions in the next projection image are automatically found around the position in the previous projection image. This procedure is repeated until the positions of BBs are detected in all projections. Subsequently, the 3D position of BBs was calculated based on the identical geometric data (positions of BBs, detector, and X-ray source) utilized in the CBCT reconstruction algorithm. The reference positions of the BBs are recorded, and the reference point is updated by a service engineer periodically maintenance once a year.

From 1 April 2022 to 26 September 2023, the Focus and CBCT precision QA were performed before the implementation of LGK treatment for patients and when maintenance is performed by a service engineer. The ICON QA tool plus was manually set on the PPS, while the geometric QA was performed fully automatically to minimize user interaction. The acquisition parameters of CBCT were: tube voltage of 90 kV, tube current of 10 mA, CT dose index of 2.5 mGy, and image resolution of 0.368 mm. The cobalt-60 sources were replaced from the end of April to the beginning of May 2023. The deviation of measured RFP from UCP and the deviation of BB’s position from reference position were analyzed from the information recorded in the machine log file. In the log file, the analysis results with and without offset values were recorded. Subsequently, the data excepting the period during the cobalt-60 source replacement operation were divided into two groups: before and after replacement. The Mann–Whitney U test was performed to measure the significance of the difference in Focus and CBCT precision QA between before and after replacement (SPSS software version 27; IBM, Armonk, NY, USA). The statistical significance was set at *p* < 0.05.

## Results

**Fig. 3:**
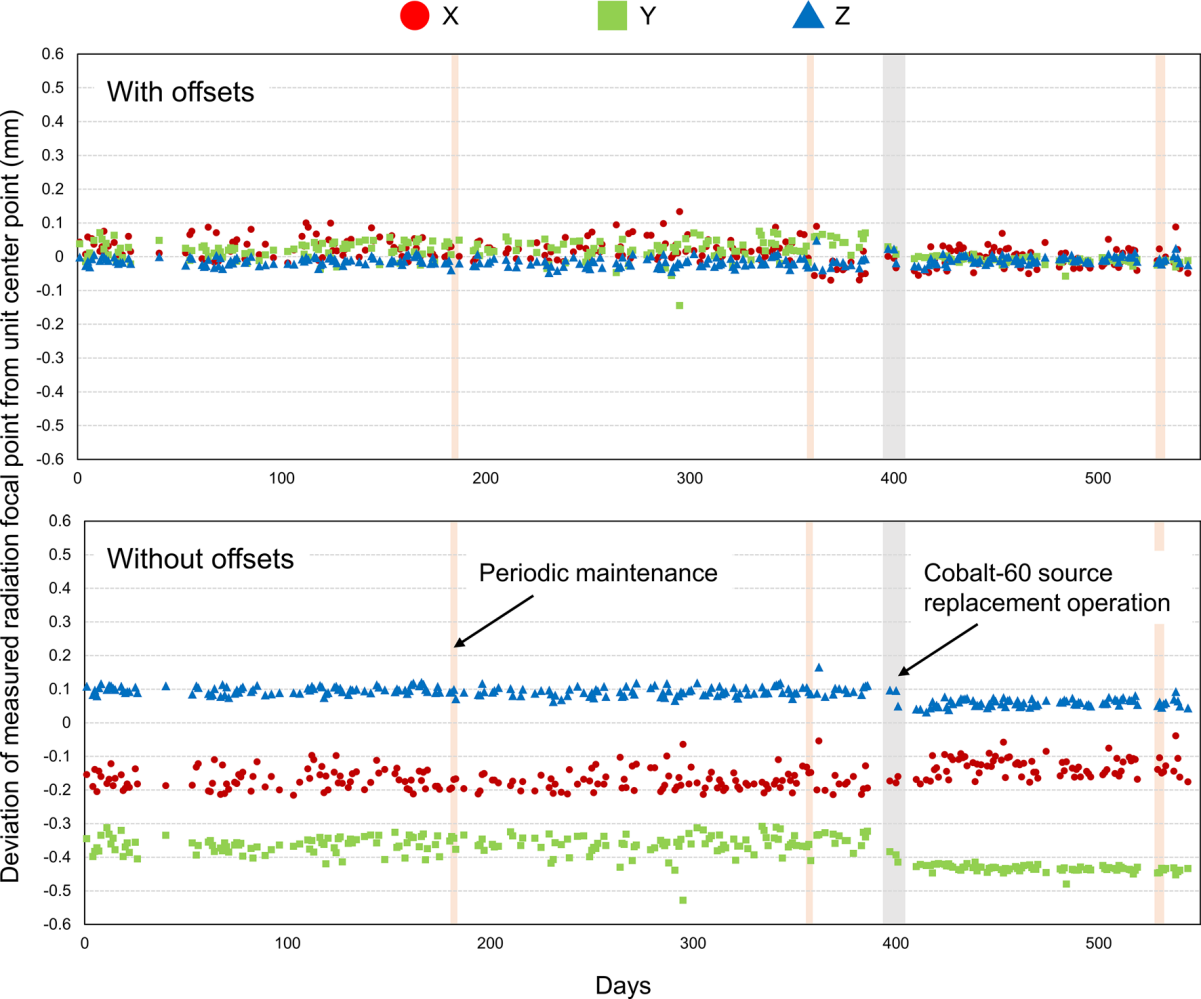
Daily deviation of measured radiation focal point from unit center point with and without offset values in three directions

During 544 days, a total of 269 Focus and CBCT precision QAs were analyzed (186 were obtained before cobalt-60 source replacement, 3 were during replacement, and 80 were after replacement). Figure 3 shows the daily deviation of measured RFP from UCP with and without offset values, and these quantitative values are summarized in Table 1. The calibrated RFP using offset values showed excellent agreement with the UCP; the deviation was within 0.1 mm in all directions on most days. The mean ± standard deviation (SD) was 0.01 ± 0.03, 0.01 ± 0.03, and −0.01 ± 0.01 mm in the *X*, *Y*, and *Z* directions, respectively. Without offset values, the deviations were largest in the Y direction with the mean ± SD of −0.38 ± 0.04 mm, and the maximum deviation was −0.53 mm. Furthermore, the significant shift of magnitude of deviation without offsets was observed after the cobalt-60 source replacement operation (*p* = 0.001). The deviations with offset values after the source replacement (0.00 ± 0.03, −0.01 ± 0.01, and −0.01 ± 0.01 mm in the *X*, *Y*, and *Z* directions, respectively) were significantly (*p* = 0.001) smaller than those before the replacement (0.02 ± 0.03, 0.02 ± 0.01 and −0.02 ± 0.01 mm in the *X*, *Y*, and *Z* directions, respectively).

**Table 1:** Summary of daily deviation of measured radiation focal point from unit center point

Calibration	Period	*X* (mm)				*Y* (mm)				*Z* (mm)			
		Mean	SD	Min	Max	Mean	SD	Min	Max	Mean	SD	Min	Max
With offsets	All (*n* = 269)	0.01	0.03	−0.07	0.13	0.01	0.03	−0.15	0.08	−0.01	0.01	−0.05	0.05
	Before replacement (*n* = 186)	0.02	0.03	−0.07	0.13	0.02	0.03	−0.15	0.08	−0.02	0.01	−0.05	0.05
	After replacement	0.00	0.03	−0.06	0.09	−0.01	0.01	−0.06	0.01	−0.01	0.01	−0.04	0.03
	*p* value (before vs. after)	0.001				0.001				0.001			
Without offsets	All (*n* = 269)	−0.16	0.03	−0.22	−0.04	−0.38	0.04	−0.53	−0.31	0.08	0.02	0.03	0.17
	Before replacement (*n* = 186)	−0.17	0.03	−0.22	−0.05	−0.36	0.03	−0.53	−0.31	0.10	0.01	0.06	0.17
	After replacement (*n* = 80)	−0.13	0.03	−0.18	−0.04	−0.43	0.01	−0.48	−0.42	0.06	0.01	0.03	0.09
	*p* value (before vs. after)	0.001				0.001				0.001			

**Fig. 4:**
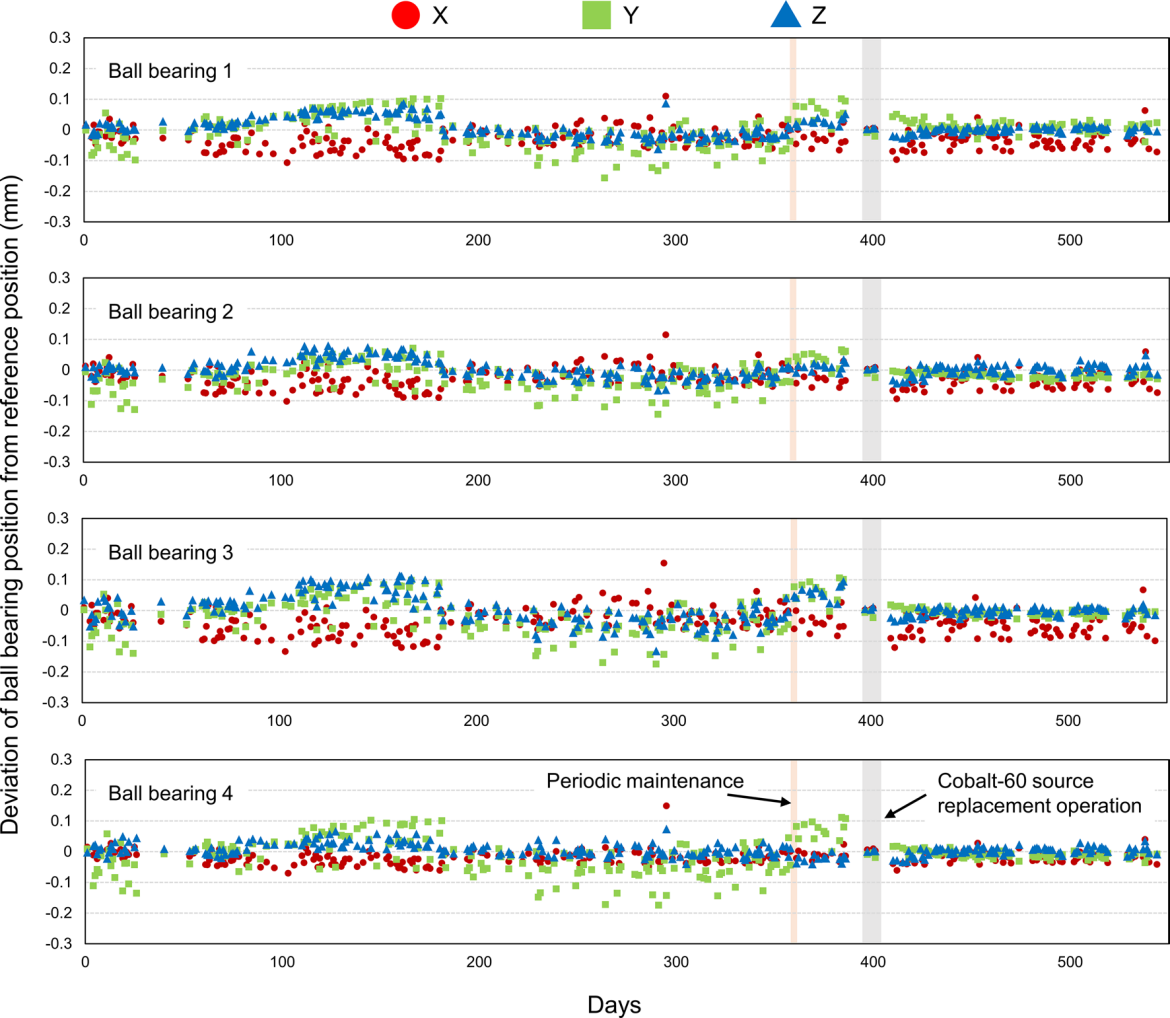
Daily deviation of ball bearings positions from reference position

Figure 4 shows the daily deviation of BB positions from the reference position, and these quantitative values are summarized in Table 2. The deviations were similar in all BBs, and the daily BB positions were matched with the reference positions; the deviations for all BBs were within 0.1 mm in all directions on most days. The overall mean ± SD of four BBs were −0.03 ± 0.03, −0.01 ± 0.05, and 0.01 ± 0.03 mm in the *X*, *Y*, and *Z* directions, respectively. The maximum deviation of −0.18 mm was observed in the *Y* direction for BB #3. There was no significant difference in deviation of BB positions between before and after the source replacement (*p* > 0.05).

**Table 2:** Summary of daily deviation of ball bearings positions from reference position

Ball bearings	Period	*X* (mm)				*Y* (mm)				*Z* (mm)			
		Mean	SD	Min	Max	Mean	SD	Min	Max	Mean	SD	Min	Max
# 1	All (*n* = 269)	−0.03	0.03	−0.11	0.11	0.00	0.05	−0.16	0.10	0.01	0.03	−0.06	0.09
# 2	All (*n* = 269)	−0.03	0.03	−0.10	0.12	−0.02	0.04	−0.14	0.07	0.00	0.03	−0.07	0.08
# 3	All (*n* = 269)	−0.04	0.04	−0.13	0.15	−0.02	0.05	−0.18	0.11	0.01	0.04	−0.13	0.11
# 4	All (*n* = 269)	−0.02	0.02	−0.07	0.15	−0.01	0.05	−0.17	0.11	0.01	0.02	−0.04	0.07
Overall	All (*n* = 269)	−0.03	0.03	−0.10	0.13	−0.01	0.05	−0.16	0.10	0.01	0.03	−0.06	0.08
	Before replacement (*n* = 186)	−0.03	0.03	−0.10	0.13	−0.01	0.06	−0.16	0.10	0.01	0.03	−0.06	0.08
	After replacement (*n* = 80)	−0.03	0.03	−0.09	0.06	−0.01	0.01	−0.03	0.02	0.00	0.01	−0.03	0.03
	*p* value (before vs. after)	0.390				0.137				0.092			

## Discussion

This study demonstrated the long-term geometric accuracy of RFP and CBCT for the ICON LGK system. Currently, three main types of LGK systems are used in clinical practice: the MODEL C [11], introduced in 1999; the PERFEXION [12], introduced in 2006; and the ICON, introduced in 2015. In each system, the UCP, the RFP, and the CBCT (if applicable) are precisely aligned when the LGK system is installed for the first time. Because LGK treatment utilizes a highly steep dose distribution, misalignment due to aged deterioration or incorrect alignment of instruments can directly affect the effectiveness of the patient’s treatment.

Traditionally film measurement with high spatial resolution is performed to confirm RFP and UCP agreement. Maitz et al. used a specially designed tool, in which a pin pierced a very small hole indicating the UCP, for film irradiation, and the coincidence of UCP and RFP was confirmed [13]. Alternatively, Maraghechi et al. utilized a MicroDiamond detector, which has 0.004 mm^3^ active volume, and demonstrated that the coincidence between the UCP and RFP was less than 0.3 mm for the ICON system [14]. In TG 178, the tolerance of Focus precision QA using the vendor-provided QA tool plus is ≤ 0.2 mm for PERFEXION and ICON LGK systems, and the deviation between the UCP and RFP should not change considerably from 1 day to the following day [6]. Our study demonstrated that the accuracy of Focus precision QA was found to be within tolerance over a long-term period when the calibration between UCP and RFP was appropriately performed. The required offset values were different in each direction (Fig. 3), and the magnitude of the offset value was slightly but significantly changed before and after the replacement operation. The reason of the slight change in offset value may be the extensive work required. The fact that significant differences were observed even when the offset value was used may be due to the skill of the engineer who determined the optimal offset value or accurately positioned the LGK system. The half-life period of the cobalt-60 sources is approximately 5.3 years, and thus, periodic source replacement operation (5–7 years) is required due to prolonged treatment time. Our data are valuable because there are not many ICON devices that have reached the time of source replacement. To replace the sources, the PPS, cables, and covers surrounding the shielding are completely removed as shown in Fig. 1, and this extensive operation may affect the offset value. In this study, the deviation became smaller after the source replacement, but it is possible that it could become larger. Careful QA to ensure proper calibration after source replacement may be worthwhile.

When installing the ICON system, the establishment of a transformation between the reconstructed image space in CBCT and the Leksell coordinate space is achieved by the manufacturer. Thereafter, the users need to perform the CBCT Precision QA to ensure the accuracy of dose delivery of CBCT-based treatment, and the tolerance is ≤ 0.4 mm. AlDahlawi et al. demonstrated that the mean ± SD of the CBCT precision QA test was 0.12 ± 0.04 mm and the deviation was below tolerance for 2 years [15]. Our study supports their data that the geometric accuracy of the CBCT equipped with the ICON system is guaranteed over time, and we provided a new finding that the geometric accuracy of CBCT before and after source replacement was unchanged. This may be explained by the fact that the deviation of CBCT geometry was calculated from the reference positions of the BBs not from the LGK coordinate system.

Several limitations are included in our study. First, we focused on the geometric accuracy of the RFP and CBCT, although there are many QA tests that should be performed such as radiation and patient safety tests, mechanical checks, and dosimetric tests [6]. Second, the deviations can vary depending on the systems, so a multi-center study is expected. We consider that the daily Focus and CBCT precision QA will continue to be necessary for LGK system [6], as it is usually treated with 0 mm margins. However, in the future, QA may be simplified with a scheduled interval if it can be shown that the accuracy of LGK at multiple facilities can be guaranteed. Finally, this study evaluated the deviation of RFP with the collimator size of 4 mm, while the ICON system can utilize the size of the collimator from 4 to 16 mm accounting for the target size. The magnitude of deviation might be varied depending on the collimator size.

In conclusion, the geometric quality assurance of RFP and CBCT for the ICON LGK system is guaranteed within a tolerance reported in AAPM TG 178 for a long-term period. The deviation of RFP was slightly but significantly changed between before and after the cobalt-60 source replacement, and careful QA may be required after the operation.
